# 21q22 amplification detection in three patients with acute myeloid leukemia: cytogenomic profiling and literature review

**DOI:** 10.1186/s13039-022-00606-0

**Published:** 2022-07-07

**Authors:** Emily M. Kudalkar, Changlee Pang, Mary M. Haag, Daniel A. Pollyea, Manali Kamdar, Gang Xu, Meng Su, Billie Carstens, Karen Swisshelm, Liming Bao

**Affiliations:** 1 Colorado Genetics Laboratory, Department of Pathology, School of Medicine University of Colorado Anschutz Medical Campus, Aurora, CO USA; 2 Department of Pathology, School of Medicine University of Colorado Anschutz Medical Campus, Aurora, CO USA; 3 Division of Hematology, Department of Medicine, School of Medicine University of Colorado Anschutz Medical Campus, Aurora, CO USA; 4 Department of Pathology, Presbyterian St. Luke Medical Center, Denver, CO USA; 5 Sema4 OpCo Inc, Stamford, CT USA

**Keywords:** Acute myeloid leukemia, Chromosome 21q22, Amplification, Cytogenomic, Outcomes

## Abstract

**Background:**

21q22 amplification is a rare cytogenetic aberration in acute myeloid leukemia (AML). So far, the cytogenomic and molecular features and clinical correlation of 21q22 amplification in AML have not been well-characterized.

**Case presentation:**

Here, we describe a case series of three AML patients with amplified 21q22 identified by fluorescence in situ hybridization using a *RUNX1* probe. Two of these patients presented with therapy-related AML (t-AML) secondary to chemotherapy, while the third had de novo AML. There was one case each of FAB M0, M1 and M4. Morphologic evidence of dysplasia was identified in both t-AML cases. Phenotypic abnormalities of the myeloblasts were frequently observed. Extra copies of 21q22 were present on chromosome 21 and at least one other chromosome in two cases. Two showed a highly complex karyotype. Microarray analysis of 21q22 amplification in one case demonstrated alternating levels of high copy number gain split within the *RUNX1* locus at 21q22. The same patient also had mutated *TP53*. Two patients died at 1.5 and 11 months post-treatment, while the third elected palliative care and died within 2 weeks.

**Conclusions:**

Our results provide further evidence that 21q22 amplification in AML is associated with complex karyotypes, *TP53* aberrations, and poor outcomes. Furthermore, we demonstrate that 21q22 amplification is not always intrachromosomally localized to chromosome 21 and could be a result of structural aberrations involving 21q22 and other chromosomes.

## Introduction

Acute myeloid leukemia (AML) is a genetically heterogeneous disease including several distinct subtypes based on cytogenetic and molecular characteristics [1, 2]. Chromosomal and molecular genetic characteristics are a major feature for risk stratification to predict outcomes and guide treatment selection [3]. Although 5-year survival of adult AML has improved in the past decades due to advances in treatment, including targeted therapies and risk-adapted regimens, more than two-thirds of AML patients do not survive beyond 5 years and most relapse [4]. Identification of additional biomarkers as new risk factors will help refine risk stratification and would have the potential to improve AML outcomes.

Genomic segmental or locus amplifications are rare in AML. They may be present in forms including homogeneously staining regions (hsr), double minutes (dmin), ring chromosomes, and marker chromosomes, resulting in a high copy number of oncogenes that are usually associated with adverse prognosis [5]. The two most commonly amplified genes in AML are *MYC* and *KMT2A(MLL)*. Amplification of *KMT2A* (located at 11q23) in AML has been reported in patients with older age, complex karyotypes, *TP53* aberrations, and inferior outcomes [6]. More recently, amplification of 21q22, defined as five or more copies per cell, has emerged as a rare cytogenomic aberration in AML. Gain of 21q22 has been reported in a limited number of AML patients, primarily case reports of adults featuring complex karyotypes [7–14]. The largest reported cohort contained 13 patients and the authors observed that 21q22 amplification was associated with reduced survival [14]. But the majority of other studies reported in the literature lack outcome information, rendering the clinical significance of this aberration uncertain. Most of the 21q22 aberrations were identified by G-banding karyotyping and/or fluorescence in situ hybridization (FISH), and only a few were characterized at the genomic level using high resolution genomic approaches such as chromosomal microarray and sequencing [12, 13]. A recent study from Nguyen and colleagues showed that the entire *RUNX1* gene may not always be included in 21q22 amplification, and as such, the molecular genomic features of 21q22 amplification in AML remain to be determined [12]. Due to the rarity of this abnormality, there is a need to further assess clinical correlation of 21q22 amplification in AML.

Here, we report a study aimed to characterize the cytogenetic, pathological, and clinical features of three AML patients with 21q22 amplification. Results from our study show that 21q22 amplification in AML may be present either in cluster or scattered across the genome and may be associated with a complex karyotype. AML with 21q22 amplification is often associated with treatment-related disease and correlated with inferior outcomes. Moreover, our microarray studies demonstrate that the full-length *RUNX1* locus is not uniformly amplified in 21q22 amplification.

## Materials and methods

### Patients

A total of 1291 new AML cases were processed at the Colorado Genetics Laboratory of the Department of Pathology at the University of Colorado Anschutz Medical Campus between 2005 and 2021. All cases were analyzed for *RUNX1*/*RUNX1T1* rearrangement derived from t(8;21)(q21.3;q22) translocation or its variants using the dual fusion Vysis LSI *RUNX1*/*RUNX1T1* FISH probe (Abbott Molecular, Green Oaks, IL). 21q22 amplification was identified by this method in 3 cases. Clinical information was obtained by medical record review.

### Morphologic and Immunophenotyping analyses

The morphologic features of bone marrow aspirates and core biopsies were reviewed for each case. The cases were classified according to the 2016 revision of WHO classification of acute myeloid leukemia, as well as the French-American-British (FAB) classification. Each case was examined for specific morphological features including presence and lineage of dysplasia. Available immunophenotype by immunohistochemistry and/or flow cytometry was reviewed in each case.

### Cytogenetic and FISH studies

G-banding karyotyping and FISH studies were performed according to standard procedures [15]. The following FISH probes were used to detect common AML cytogenetic aberrations: 5p15.2/5q31*(EGR1*), CEP7/7q31(D7S486), *RUNX1*/*RUNX1T1*, *KMT2A(MLL)*, *PML*/*RARA* and *CBFB* from Abbott Molecular (Green Oaks, IL), *TP53*, *MECOM*/3q26 and *DEK*/*NUP214* from MetaSystems (Altlussheim, Germany). In this study, 21q22 amplification was defined as five or more copies of *RUNX1* per cell by FISH, and a complex karyotype was deemed as having three or more chromosomal abnormalities. Karyotypes were described following the 2016 International System for Cytogenomic Nomenclature (ISCN) [16].

### Chromosomal microarray analysis

Bone marrow samples were prepared by extracting DNA using the Maxwell RSC Blood DNA kit (Promega, Madison, WI). Microarray analysis was performed using the Infinium CytoSNP-850K v1.2 BeadChip (Illumina, San Diego, CA). DNA was amplified, enzymatically fragmented, and hybridized to probes on the array following the manufacturer’s instruction. Array images were scanned using the GeneChip Scanner 3000 7G (Illumina, San Diego, CA). Copy number variation and B-allele frequency were analyzed using the BlueFuse Multi software (Illumina, San Diego, CA). Genomic coordinates refer to the human genome GRCh37/hg19 build.

### Molecular genetic analysis

DNA was extracted from the bone marrow aspirates and paired normal tissue (fingernail), and was analyzed for mutations in an AML panel of 49 genes (*ASXL1*, *BCOR*, *BCORL1*, *BRAF*, *CALR*, *CBL*, *CEBPA*, *CSF3R*, *CXCR4*, *DDX41*, *DNMT3A*, *ETV6*, *EZH2*, *FLT3*, *GATA1*, *GATA2*, *HRAS*, *IDH1*, *IDH2*, *JAK1*, *JAK2*, *JAK3*, *KDM6A*, *KIT*, *KMT2A*, *KRAS*, *MPL*, *MYD88*, *NF1*, *NOTCH1*, *NPM1*, *NRAS*, *PHF6*, *PPM1D*, *PTEN*, *PTPN11*, *RAD21*, *RUNX1*, *SETBP1*, *SF3B1*, *SH2B3*, *SRSF2*, *STAG2*, *STAT3*, *TET2*, *TP53*, *U2AF1*, *WT1*, *ZRSR2*). Samples were processed using a custom hybridization-probe capture method (IDT) and run on the Illumina NovaSeq platform. Data was processed using a custom bioinformatics pipeline and analyzed with Fabric Genomics software. Paired normal tissue was used to determine the germline status of each variant. Variant interpretation was based on the ACMG-AMP guidelines for somatic variants [17].

## Results

### Karyotype and FISH results

**Fig. 1:**
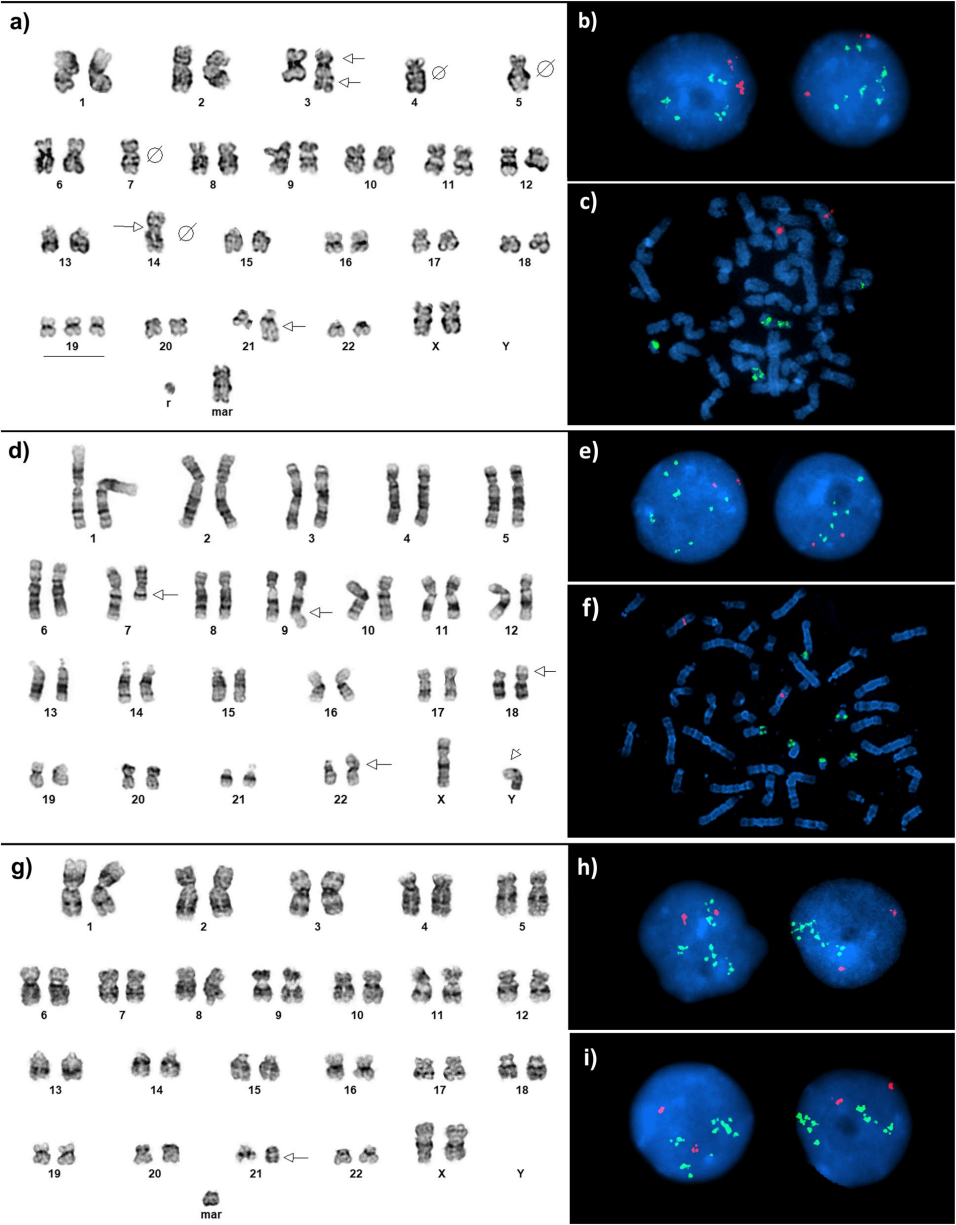
Representative karyotypes and FISH findings of patients with 21q22 amplification. Patient 1: **a** karyotype, **b** interphase FISH showing 5–10 copies of *RUNX1* and **c** metaphase FISH, with *RUNX1* localized to hsr(21), chromosome 14, and a marker chromosome. Patient 2: **d** karyotype, **e** interphase FISH showing 4–15 copies of *RUNX1* and **f** metaphase FISH with *RUNX1* localized to Yp, 5q, 9q, and 22p for this metaphase. Patient 3: **g** karyotype and **h**–**i** interphase FISH, showing 10 copies of *RUNX1*. Arrows denote structural aberrations and circle with slash denote loss of chromosome. Mar = marker chromosome, r = ring chromosome. Dual-colored *RUNX1*(green)/*RUNX1T1*(red) probe with nuclei visualized using DAPI (blue)

Three AML patients with 21q22 amplification were identified by *RUNX1* FISH, representing approximately 0.23% of AML cases tested at the lab during the study period. All cases showed five or more copies of *RUNX1* probe signals. Patient 1 displayed a highly complex karyotype of 45,XX,der(3)inv(3)(p25q25)add(3)(q11.2),-5,-7,+12,-14,add(14)(p11.2),+19,hsr(21)(q22)[3]/45,sl,-4,-12,+r,+mar[15]/46,sdl1,+8[2] in three clones, each having monosomy 5 and 7 and an hsr on chromosome 21 (Fig. 1a). Interphase FISH revealed a total of 5–10 copies of *RUNX1* signal per cell and sequential metaphase FISH identified several signals clustered within the hsr(21) and one *RUNX1* signal each on chromosome 14 and a marker chromosome of unknown origin (Fig. 1b–c). Patient 2 also showed a complex karyotype in multiple clones: 46,XY,del(7)(q22)[1]/46,idem,-Y,+der(Y)t(Y;21)(p11.3;q22.1),der(5)t(5;21)(q35;q22.1)[5]/46,idem,-Y,+der(Y)t(Y;21)(p11.2;q22.1)dup(21)(q22.1q22.3),der(9)t(9;21)(q34;q22.1)dup(21)(q22.1q22.3),der(18)t(18;21)(p11.3;q22.1)[14] (Fig. 1d). The abnormalities included 5q and 7q deletions and multiple structural rearrangements involving duplication of a 21q22 segment or dup(21)(q22) (Fig. 1d). Interphase FISH using *RUNX1*/*RUNX1T1* revealed cells with 4–15 copies of *RUNX1* signals and metaphase FISH showed the *RUNX1* signals were scattered across multiple chromosomes: Yp, 5q, 9q, 18q, and 22p (Fig. 1e–f). Patient 3 had a karyotype of 46,XX,?r(21)(q11.2q22)[6]/47,sl,+mar[10]/46,XX[4] with a ring-like abnormal chromosome 21 in two clones (Fig. 1g). Interphase FISH showed multiple clustered *RUNX1* signals, likely present on the ring chromosome 21 (Fig. 1h–i) (metaphase FISH was not performed).

### Bone marrow morphologic and immunophenotyping features

Patient 1 was noted to have severe pancytopenia with 3% circulating blasts. Subsequent bone marrow examination revealed a normocellular marrow with 55% phenotypically abnormal myeloblasts. The blasts were medium to large in size and had round to irregular nuclear outlines and dispersed nuclear chromatin with prominent nucleoli. There was minimal maturation in the myeloid lineage with mild dysgranulopoiesis. By flow cytometry immunophenotyping, the blasts expressed bright CD34, CD117, decreased CD38, CD13, variable CD33, variable HLA-DR, variable CD7 and partial CD56. The case was diagnosed as t-AML by WHO classification and subtype M1 according to FAB classification.

Patient 2 presented with severe thrombocytopenia, macrocytic anemia and 4% circulating blasts at an outside institution. Bone marrow examination revealed approximately 35% blasts and blast equivalents (promonocytes) and mild dysmegakaryopoiesis. By immunohistochemistry, the blasts/blast equivalents were positive for CD34, CD117 and negative for CD61. Flow cytometry data were not available. The findings were diagnostic for t-AML by WHO classification and FAB subtype M4.

Patient 3’s initial bone marrow biopsy showed 67% blasts without morphologic evidence of myeloid differentiation. By flow cytometry, the blasts expressed CD34, HLA-DR, CD117, increased CD33, CD11b, TdT and partial dim CD79a without expression of MPO. The case was diagnosed as AML, NOS by WHO classification or FAB subtype M0.

### Chromosomal microarray findings

**Fig. 2:**
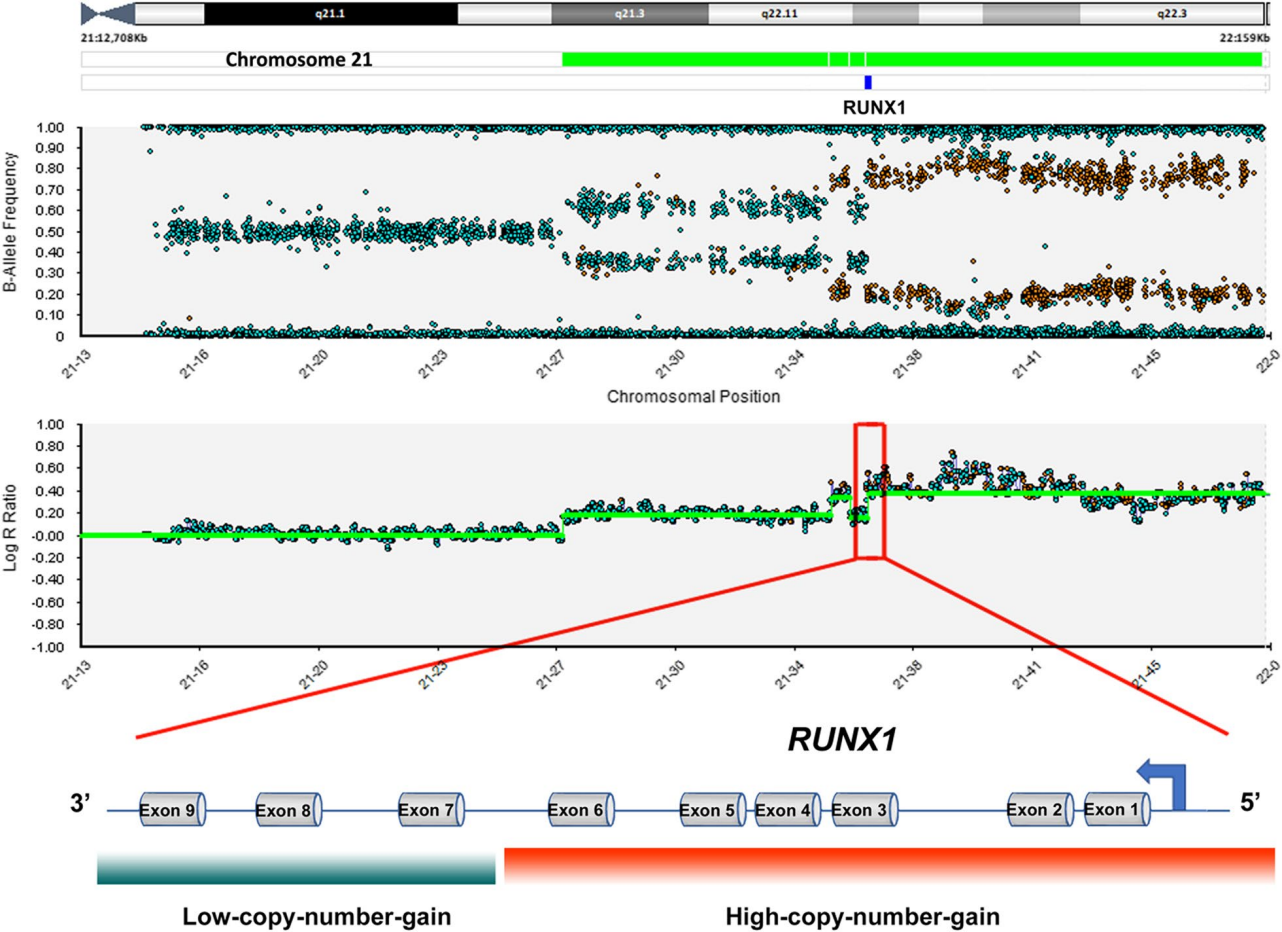
Focused chromosomal microarray analysis of 21q22 amplification observed in Patient 1. B-allele frequency and Log R ratio copy number plot of chromosome 21 demonstrate multiple regions of various copy number gains. One breakpoint lies within *RUNX1*, with high-copy-number gain (~ 3 to 6 copies) of the 5′ region of the gene containing exons 1–6 and low-copy-number gain of exons 7–9 (~ 3 copies)

Chromosomal microarray was performed on DNA extracted from bone marrow of patient 1 and the analysis was focused on chromosome 21. The results showed alternating levels of gain at 21q21.3q22.3 (chr21:27,129,093–48,100,155), with high-copy-number (~ 3 to 6 copies) at distal 21q22.12q22.3 (chr21:36,230,819–48,100,155) compared to more proximal 21q22.12 segment (~ 3 copies, chr21:27,129,093–35,134,557) (Table 1). The high-copy-number gain on 21q21.3q22.3 covered the 5′ *RUNX1* that includes non-coding exons 1–2 and coding exons 3–6, (NM_001754.4) while the rest of the *RUNX1* gene (exons 7–9) is within the low-copy-number gain segment (Fig. 2). This finding was confirmed by FISH using *RUNX1* break-apart probes targeting the sequences flanking the *RUNX1* locus, with ~ 3 copies and 4–7 copies of probe signals corresponding to 3′ and 5′ portions of the *RUNX1* locus, respectively (data not shown).

**Table 1:** Results from focused microarray analysis of 21q22 amplification in Patient 1

Cytogenetic band	Genomic coordinates (GRCh37/hg19)	Size	Copy number
21q21.3–21q22.11	Chr21:27,129,093–35,134,557	8.0 Mb	~ 3
21q22.11–21q22.11	Chr21:35,138,326–35,725,729	587.4 Kb	~ 3 to 6
21q22.11–21q22.12	Chr21:35,726,226–36,228,360	502.1 Kb	~ 3
21q22.12–21q22.3	Chr21:36,230,819–48,100,155	11.9 Mb	~ 3 to 6

### Molecular genetics findings

Mutational analysis was performed on Patient 1 and showed two somatic pathogenic *TP53* variants, NM_001126112.2: c.1024C > T, [p.Arg342Ter] and c.817C > T, [p.Arg273Cys]. No mutations were identified in the other 48 myeloid-related genes analyzed, including *RUNX1*, *FLT3*, *NPM1*, *WT1*, *CEBPA*, and *IDH1*.

### Clinical outcomes

Two patients had a prior history of malignancy: Patient 1 (77 years, female) with follicular lymphoma, and Patient 2 (26 years, male) with Hodgkin lymphoma; both developed therapy-related AML (t-AML). Patient 3 (32 years, female) had no history of malignancy and was diagnosed with AML when she was 25 weeks pregnant. Patient 1 was treated with venetoclax/azacitidine and died 1.5 months after diagnosis. Patient 3 deferred treatment until after delivery and then received “7 + 3” induction chemotherapy (idarubicin/cytarabine). She then went on to receive an allogeneic stem cell transplantation in the setting of persistent measurable residual disease and died of disease relapse 11 months after diagnosis. Patient 2 elected palliative care and received no treatment for AML; he died 2 weeks after diagnosis (Table 2).

**Table 2:** Clinical and cytogenomic and molecular characteristics of patients with AML featured with 21q22 amplication

Pt	Age/sex	Diagnosis	Prior malignancy	Karyotype	*RUNX1* FISH copy number and localization	Treatment and outcome	Molecular findings
1	77/F	t-AML	Follicular Lymphoma	45,XX,der(3)inv(3)(p25q25)add(3)(q11.2),-5,-7,+12,-14,add(14)(p11.2),+19,hsr(21)(q22)[3]/45,sl,-4,-12,+r,+mar[15]/46,sdl1,+8[2]	*RUNX1*x5–10: present on marker, hsr(21), add(14)	Venetoclax/Azacitadine. Died within 1.5 months	Bi-allelic *TP53* pathogenic mutations
2	26/M	t-AML	Hodgkin Lymphoma	46,XY,del(7)(q22)[1]/46,idem,-Y,+der(Y)t(Y;21) (p11.3;q22.1),der(5)t(5;21)(q35;q22.1)[5]/46,idem,-Y,+der(Y)t(Y;21)(p11.2;q22.1)dup(21)(q22.1q22.3), der(9)t(9;21)(q34;q22.1)dup(21)(q22.1q22.3),der(18)t(18;21)(p11.3;q22.1)[14]	*RUNX1*x4–15: Yp, 5q, 9q, 18q and 22p	Palliative care. Died within 2 weeks	n/s
3	32/F	AML	None	46,XX,?r(21)dup(21)(q11.2q22)[6]/47,sl,+mar[10]/46,XX[4]	*RUNX1*x10	Idarubicin/cytarabine, double cord transplant. Died 11 months	n/s

*n*/*s* not studied

## Discussion

21q22 amplification by *RUNX1* FISH is rare in AML with a reported prevalence of 0.1% [14]. Most were identified by cytogenetically visible abnormal 21q or “incidental” findings by *RUNX1* FISH [7–14]. Due to this limitation, the true incidence of 21q22 amplification in AML remains elusive. Most were reported in adults with complex chromosomal abnormalities. Fewer cases reported thus far had accompanying clinical outcomes, leaving the impact of 21q22 amplification on AML outcome uncertain.

There is limited understanding of cytogenomic features of 21q22 amplification in AML. In this study, the high-resolution microarray study of Patient 1 showed high-copy-number gain (~ 3 to 6 copies) of the 21q22 segment (11.9 Mb, chr21:36,230,819–48,100,155) covering the 5′ portion of the *RUNX1* locus compared to the smaller low-copy-number gain of the 21q22 segment in the 3′ portion of *RUNX1* (~ 3 copies, 502.1 kb, chr21: 35,726,226–36,228,360). A similar observation was reported by Nguyen and colleagues who described an AML patient with 21q22 amplification covering a portion of the 5′ region of *RUNX1* and extending to the 21q terminus, albeit with different breakpoints than the ones observed in our patient [12]. In their microarray analysis of 33 AML cases with 21q22 amplification, defined as six or more copies of *ERG* by FISH, Weber and colleagues reported the *ERG* locus as the commonly amplified in 21q22 region while the *RUNX1* locus was amplified in only about half of the cases [13]. This is further supported by the observation that *RUNX1* expression is not upregulated in AML with amplified *RUNX1* identified by *RUNX1* FISH [9]. Our report is the second study to show that 21q22 amplification identified by *RUNX1* FISH does not include the entire *RUNX1* locus [12] and the *ERG* locus is within the high-copy-number gain region. The *RUNX1* FISH approach is likely to lead to an underestimation of the incidence of 21q22 amplification in AML because it would not identify those cases that do not involve the *RUNX1* locus. Xie et. al reported a prevalence of 0.1% using *RUNX1* FISH to ascertain 21q22 amplification in their AML cohort and using the same approach, we found a frequency of 0.2% in our AML cohort, while Weber et al. described a higher prevalence of 0.7% using *ERG* FISH [13, 14].

*ERG* encodes a proto-oncogenic transcription factor expressed in hematopoietic stem cells including those present in AML and myelodysplastic syndrome (MDS) [18] *ERG* overexpression has been shown to be an adverse biomarker for cytogenetically normal AML [19, 20]. Moreover, Carmichael and colleagues showed that *Erg* overexpression in mice induced the development of an erythro-megakaryocytic leukemia [21]. Weber et al. demonstrated that *ERG* overexpression is correlated with *ERG* high-copy-number gain, indicating that amplification may result in upregulation of *ERG* expression [13]. *RUNX1* truncating and frameshift mutations have been reported in myeloid neoplasms, supporting its role as a tumor suppressor [22]. Co-existence of *RUNX1* mutations and *ERG* amplification was observed in 25% of AML cases with 21q22 amplification [13]. However, whether the *RUNX1* mutation cooperates with *ERG* amplification to promote AML leukemogenesis remains unclear at the present time.

In this study, two patients, Patients 1 and 3, had an hsr on an abnormal chromosome 21. These results are in agreement with the observations by others who described hsr(21) as a common form of 21q22 amplification in AML [10, 12, 14]. Interestingly, Patients 1 and 2 in our study displayed 21q22 gain scattered across several different chromosomes. The *RUNX1* FISH probe signals were dispersed in interphase nuclei and metaphase chromosomes rather than as a clustering pattern seen in hsr(21). Jain and colleagues described AML in a 6-year-old child who had an hsr(21) with additional 21q22 material inserted into chromosome 2q31 [10]. Similar observations were also documented in other AML cases [8, 11]. These findings demonstrate that 21q22 amplification in AML may present as either gain of 21q22 clustering on chromosome 21 and/or the presence of multiple derivative chromosomes harboring the 21q22 segment.

Previous mutational analysis revealed that mutant *TP53* is common in AML with 21q22 amplification and accordingly, Patient 1 in our study also harbored dual *TP53* mutations [8, 14]. Nguyen et al. similarly found a *TP53* deletion detected by FISH in an AML patient with 21q22 amplification [12]. *TP53* plays a role in maintaining genomic stability and has been linked to chromothripsis [23], therefore, it may be an early event resulting in 21q22 amplification.

So far, there is limited information on clinical characteristics and outcomes in AML with 21q22 amplification [7, 9, 10, 14]. In this study, two patients were young adults of 26 and 32 years at diagnosis, respectively, and the third patient was 77 years old. A highly complex karyotype with multiple clones was observed in two patients who had a prior history of malignancy. These patients presented with different primary malignancies (follicular lymphoma and Hodgkin lymphoma) and were treated with different therapeutics, despite both developing t-AML. Our results concur with other reports which describe 21q22 amplification in patients with t-AML, who also displayed various primary malignancies and received different treatments [8, 12, 14]. Of the limited number of AML patients with 21q22 amplification whose clinical outcomes were available, most had worse outcomes with median survival of 3.2 months reported in one study [14]. In the present study, two patients did not survive beyond 1.5 and 11 months, respectively, and the third who elected palliative care died within 2 weeks of diagnosis. Because *TP53* aberrations and complex karyotypes are known adverse risk features for AML [24, 25] and 21q22 amplification is not always seen in de novo AML, additional studies of larger cohorts are necessary to further determine whether 21q22 amplification is a new AML adverse biomarker for worse outcomes or just a secondary or passenger change as a result of co-existence of a complex karyotype, chromosome 7 abnormalities, or *TP53* aberrations.

This case series study has several limitations. First, this is a small cohort of three patients, rendering it difficult to adequately assess whether 21q22 amplification is a poor prognostic factor independent from other known adverse risk factors in AML, including complex karyotype and chromosome 7 abnormalities present in 2 of 3 our patients. Second, we were not able to perform gene expression studies on the *ERG* gene due to the lack of materials. Third, molecular studies were only done on one patient, and lastly, high resolution genomic analysis of 21q22 amplification using chromosomal microarray was performed on one case, again due to the lack of materials. Future large international collaborations are necessary to further characterize the genomic features and the prognostic significance of 21q22 amplification in AML.

In summary, we report the cytogenomic features, histopathologic findings, and clinical outcomes in three adult AML patients with 21q22 amplification. Our results show that AML with 21q22 amplification is associated with a complex karyotype, poor prognosis, and occurs in both de novo AML and t-AML. 21q22 amplification may result from either a cluster on a single chromosome in the form of hsr(21) or multiple 21q22 rearrangements scattered across different chromosomes. Findings from this study support that the *RUNX1* locus is not a primary target of 21q22 amplification in AML.
